# Highlighting the Multifaceted Role of Orai1 N-Terminal- and Loop Regions for Proper CRAC Channel Functions

**DOI:** 10.3390/cells11030371

**Published:** 2022-01-22

**Authors:** Christina Humer, Christoph Romanin, Carmen Höglinger

**Affiliations:** Institute of Biophysics, Johannes Kepler University Linz, Gruberstrasse 40, 4020 Linz, Austria; christina.humer_1@jku.at (C.H.); christoph.romanin@jku.at (C.R.)

**Keywords:** calcium, Orai1, CRAC channel, N-terminus, Loop2

## Abstract

Orai1, the Ca^2+^-selective pore in the plasma membrane, is one of the key components of the Ca^2+^release-activated Ca^2+^ (CRAC) channel complex. Activated by the Ca^2+^ sensor in the endoplasmic reticulum (ER) membrane, stromal interaction molecule 1 (STIM1), via direct interaction when ER luminal Ca^2+^ levels recede, Orai1 helps to maintain Ca^2+^ homeostasis within a cell. It has already been proven that the C-terminus of Orai1 is indispensable for channel activation. However, there is strong evidence that for CRAC channels to function properly and maintain all typical hallmarks, such as selectivity and reversal potential, additional parts of Orai1 are needed. In this review, we focus on these sites apart from the C-terminus; namely, the second loop and N-terminus of Orai1 and on their multifaceted role in the functioning of CRAC channels.

## 1. Introduction

Amounting approximately 1 kg of the body weight of an adult, calcium (Ca^2+^) is the most abundant mineral present within the human body, with most of it being bound within solid structures like bones and teeth. The remaining minor fraction of soluble calcium mediates a multifaceted repertoire of physiological processes, including immunological defense, muscle contraction and neurotransmission that rely on temporally and spatially reversible increases in the concentration of free cytosolic Ca^2+^ ions [1,2,3]. Cells can harness two types of sources to raise the latter from a marginal resting value of about 100 nM by up to a factor of 10: the extracellular space, typically defined by concentrations of 1–2 mM, or specific intracellular Ca^2+^-storing organelles. Considering the latter, the endoplasmic reticulum (ER) has the largest storage capacity due to its size [2]. The establishment and maintenance of resting Ca^2+^ levels in the cell and elevations upon activation involve the coordinated interplay of various proteins. Thereby, the opening of ion channels in the plasma membrane (PM) or in the phospholipid bilayers of intracellular compartments culminates in Ca^2+^ influx into the cytosol. Such an activation of ion channels may be triggered by various signals, including changes in the transmembrane potential, mechanical stimulation or ligand binding [1,2,4,5,6]. Outstanding representatives of channels responding to the last-mentioned kind of activation stimulus are Ca^2+^ release-activated Ca^2+^ (CRAC) channels. Upon depletion of the ER, these are assembled from two different proteins that reside within separate membrane compartments: stromal interaction molecule 1 (STIM1) and Orai1. While STIM1 is present within the membrane of the ER, Orai1 forms the Ca^2+^ selective pore within the plasma membrane. For CRAC-channel activation to occur, the generation of soluble inositol 1,4,5-triphosphate (IP_3_) by phospholipase C (PLC)-mediated cleavage of the membrane phospholipid phosphatidylinositol 4,5 bisphosphate (PIP_2_) is vital. Following its release, the splitting product is captured by IP_3_ receptors of the ER-membrane and, as these function as Ca^2+^ permissive channels, they induce Ca^2+^ ions to exit the ER lumen based on the concentration gradient. The associated receding in the ER–Ca^2+^ reservoir triggers a series of conformational and spatial reorganizations of STIM1 proteins [4,7,8,9,10]. These events culminate in direct interactions between STIM1 and Orai1, finally leading to the opening of the Orai1-formed pore [11,12,13,14,15]. Overall, processes whereby the influx of Ca^2+^ from the extracellular space is triggered upon store depletion are referred to as “store-operated Ca^2+^ entry” (SOCE), the basic concept of which was first presented by J.W. Putney in 1986 [16,17,18].

Among the ion channels reported to operate in such a store-operated manner, CRAC channels are currently the most investigated representative [18]. After STIM1 and Orai1 were identified as essential elements of the CRAC channel complex in 2005 and 2006, respectively, extensive structural and functional analysis revealed that STIM1 proteins span the ER-membrane with a single transmembrane (TM) domain. Further, STIM1 contains an EF-sterile alpha motif (EF–SAM) domain within its ER -luminal part to sense the Ca^2+^ concentration within the organelle. The latter sensing relies on the binding of Ca^2+^ ions to the EF-SAM domain in the resting state of the cell, thereby establishing an equilibrium between STIM1-bound and free Ca^2+^ that is dependent on the affinity of STIM1 for the ion and the concentration of free Ca^2+^. In turn, the ongoing equilibration between protein-bound and free Ca^2+^ forces the dissociation of the ion upon store depletion [19,20,21,22,23]. This serves as a trigger for a conformational opening of the N-terminal fold that is further relayed through the short transmembrane segment to the cytosolic STIM1 C-terminus which, folded towards the ER membrane upon rest, consequently engages a more extended conformation [9,10,12,24,25,26,27]. The cytosolic portion of STIM1 comprises three coiled coil (CC) regions of which several fragments have been identified as being sufficient to strongly activate Orai1 in a store-independent manner if expressed in isolation from the ER-luminal- and transmembrane regions, respectively. These involve the STIM1_342-448_ segment (CRAC activation domain, CAD), the somewhat shorter STIM1 Orai Activating region (SOAR, STIM1_344-442_), the coiled-coil domain containing region b9 (CCb9, STIM1_339-444_), STIM1_233-450_ or the Orai-activating small fragment (OASF, STIM1_233-474_) [11,28,29,30]. Conformational remodeling within the cytosolic segment of the Ca^2+^-sensing protein, together with the transition to ER- PM junctions where the distance among both membranes declines to 10–25 nm, allows for direct coupling with Orai1 proteins when the ER gets depleted [31,32]. Consequentially, the plasma membrane channels become trapped, co-cluster with STIM1, and the gates of the pore are finally unleashed [11,33,34,35].

The assignment of Ca^2+^ sensing functions and Orai1 interaction abilities to the different STIM1 termini is alleviated by its architecture as a single-pass transmembrane protein. However, the characterization of Orai1 in view of possible STIM1 binding sites, the relaying of protein coupling into pore opening and the deciphering of the actual gating events is comparatively intricate. Therefore, insights into the structure of Orai channels were highly desirable, some aspects of which will be reviewed below.

## 2. Orai1 Structure

The family of human Orai proteins comprises three homologs (hOrai1-3) that, in collective with the sensor proteins STIM1 and STIM2, choreograph Ca^2+^ signaling in a graded and diverse fashion [36]. Even though three murine orthologs have also been identified [37,38], herein, the simplified designations Orai1, Orai2 and Orai3 are deployed to refer to the human proteins hOrai1-hOrai3 only [22,39,40,41,42]. However, the Orai proteins share general structural features and are associated with a comparatively high degree of sequence conservation within their transmembrane domains, but they are considerable different from other Ca^2+^-selective channels [22,39,40,41,42].

Among the three Orai isoforms, Orai1 is currently the best investigated and also represents the main subject of this review, whereas a closer comparison of the structural organization and the respective physiological functions of the three Orai isoforms is provided in [42]. The primary structure of Orai1 monomers comprises 301 amino acids, which amounts to a molecular mass of 33 kDa. Leadoff studies on Orai proteins in view of hydrophobicity analysis and immunocytochemistry revealed the protein to comprise four transmembrane domains and that both of its termini reside within the cytosol. Accordingly, the second loop region linking the second and the third transmembrane segment is also cytosolic, while the first and the third loops are extracellular [22]. Although the structure of human Orai1 has not yet been resolved,, this basic architecture was supported by structures of drosophila Orai (dOrai), determined in the closed and presumably open state via crystallography and single particle cryo-electron microscopy (cryo-EM) [43,44,45]. In each of these efforts, six dOrai proteins were found to assemble to form the overall plasma membrane channel, what was startling as a tetrameric composition was widely expected in the CRAC channel community. Yet, experimental data on Orai1 is consistent with a hexameric composition of the CRAC channel pore as well so that the latter stoichiometry represents currently the mostly assumed consensus [46,47].

**Figure 1:**
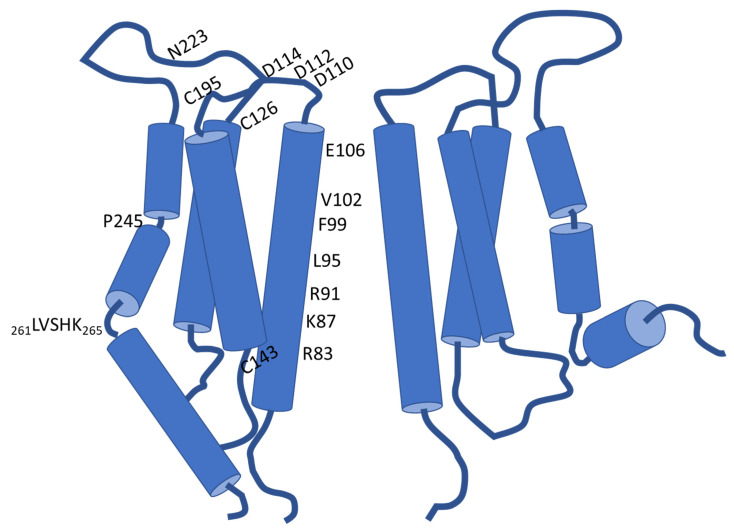
Schematic on the topology of Orai1 channels, highlighting the positions of functionally important residues. For the sake of simplicity, only two opposing Orai1 proteins are shown. The acidic residues D110/D112/D114 reside at the extracellular mouth of the pore and serve as Ca^2+^ accumulating region (CAR), while E106 defines the narrowest area along the pore and functions as a selectivity filter. Further in direction towards the cytosol, the pore is lined by the hydrophobic residues V102, F99 and L95, as well as the subsequent basic side chains of R91, K87 and R83, respectively. N223 in the third loop represents a glycosylation site, while the cysteines at the positions 126, 143 and 195, are involved in redox regulation of Orai1. P245 disrupts the continuity of the fourth transmembrane helix and may lead to constitutive activity when mutated. The five subsequent residues _261_LVSHK_265_ define the nexus domain, which is relevant for gating as well (see text for further details).

Each of the four transmembrane domains of one Orai subunit engages an α-helical structure (Figure 1). Considering the overall hexamer, the TM domains are arranged into a triad of concentric rings that enclose the rather narrow ion permeation pathway in their center. Collectively, the first transmembrane helices form the inner ring so that the chemical milieu within the pore is ruled by the properties of those TM1 residues that serve as its linings. The pore, which measures about 55 Å in length, is in this regard defined by four consecutive regions. These commence with an acidic ring at the extracellular ingress that is associated with the smallest pore diameter and serves as the selectivity filter (SF). In the case of human Orai1, this selectivity filter is primarily formed by six glutamates at position 106 (E106) [21,43,48]. Further towards the extracellular milieu from the selectivity filter is another local assembly of acidic residues, comprising D110/112/114. This arrangement was shown to be critical for elevating the concentration of Ca^2+^ in close vicinity of the SF. Consistently termed as “Ca^2+^ accumulating region (CAR)”, the acidic cluster was identified to promote permeation particularly under conditions of low extracellular Ca^2+^ levels [21,49]. Next to the selectivity filter in the direction of ion permeation, a hydrophobic segment establishes the chemical milieu within the pore throughout ~18 Å. Thereafter, a region that is lined by lysine and arginine residues and later, a wider-diameter section, delineate the remaining pore sections [43]. The hydrophobic segment (Orai1 V102, F99, L95) and the subsequent basic domain (Orai1 R91, K87, R83) are thought to function as gates that impede Ca^2+^ influx into the cytosol under resting cell conditions [45,50,51,52]. Thereby, the substitution of the charged R91 by a hydrophobic tryptophan residue is well established to give rise to severe combined immune deficiency (SCID) [22]. The concerning mutation was shown to altogether abrogate Ca^2+^ entry via Orai1 and was indeed essential in the identification of the pore-forming component of CRAC channels [22,53].

Within the triad of concentrically arranged transmembrane domains, the intercalated helical shell is composed of both the second and the third transmembrane domains of the six Orai proteins. Instead, the outermost ring is shaped by the six TM4 helices only. A proline residue in TM4 (Orai1 P245) disturbs its continuity and structures it into two segments in the resting state, while potentially getting straightened into a continuous helix upon activation [43,44,45].

As already mentioned, the first loop connecting TM1 and TM2 functions in the electrostatic attraction of Ca^2+^ based on the CAR. Contrasting this function, the other loop which faces the cell exterior (loop3) contains the sole glycosylation site of Orai1: residue N223 (see Figure 1). According to Dörr et al. (2016), this post translational modification occurs in a cell-type specific manner and is decisive for the interaction of Orai1 with carbohydrate-binding proteins (lectins), which eventually attenuate SOCE [49,54]. Moreover, cysteine residues present at the interfaces of the extracellular loops and the following transmembrane domains, C126/195, respectively, but also C143 at the beginning of the intracellular loop contribute to redox regulation of Orai1. In this regard, exposure to H_2_O_2_ imposes an inhibitory effect on SOCE. Intriguingly, analysis of the redox regulation of CRAC channels suggests inhibition to be curtailed if cells are already in a preactivated state upon H_2_O_2_ treatment, which is eventually indicative for a state-dependent modulation [55,56].

**Figure 2:**
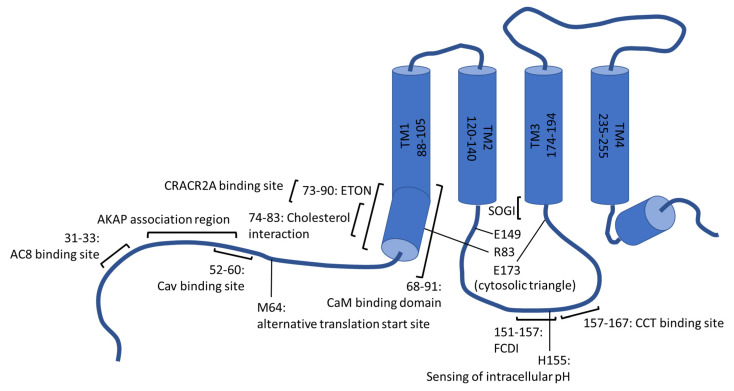
Overview on Orai1 N-terminal or loop2 regions involved in interactions with internal proteins, gating, the regulation by lipids or intracellular pH and, among others, CRAC channel inactivation. AC8: adenylyl cyclase 8, AKAP: A-kinase anchoring protein, Cav: Caveolin, CaM: Calmodulin, CCT: chaperonin-containing T-complex protein 1, CRACR2A: CRAC regulator 2A, ETON: extended transmembrane Orai1 N-terminal region, FCDI: fast Ca^2+^ dependent inactivation, SOGI: STIM1-Orai1 gating interface, TM: transmembrane.

On the cytosolic side of the plasma membrane, the first and fourth transmembrane helix reach, in strong contrast to the others, markedly into the intracellular space (20–25 Å) [43]. Considering TM1, the cytosolic protrusion is referred to as the extended transmembrane Orai1 N-terminal (ETON) region and comprises the residues 73–90 [57] (Figure 2). TM4 is followed by a further helical segment within the cytoplasm, known as M4ext. Indeed, this specific, conserved appendage was a matter of extensive research already before its structural characterization. This is explained by the mapping of a putative coiled-coil domain to the segment, which involves, in the case of Orai1, the residues 268–291 and is widely accepted to represent the main and possibly initial binding site of STIM1 [44,45,58,59,60]. Thereby, electrophysiological characterization and fluorescence resonance energy transfer (FRET)-based protein–protein interaction analysis identified the interaction between STIM1 and Orai1 to be more sensitive to mutations within the concerning Orai segment compared to the binding between STIM1 and Orai2 or Orai3. The coiled-coil motifs were consistently reported to be comparatively stronger in the case of the latter homologs [59]. Considering Orai1, the leucine residues L273 and L276 were identified by Frischauf et al. (2009) to be critical for interactions with STIM1, which was recently confirmed in a study by Baraniak and colleagues (2021) using membrane-anchored but isolated Orai1 C-terminal peptides [59,61].

With regard to dOrai-formed CRAC channels in the closed state, the C-termini of the six subunits are present in two different conformations, which are engaged in an alternating manner by adjacent proteins [43]. This demands for structural flexibility, which is accomplished by a conserved hinge region (Orai1 _263_SHK_265_) that forms, together with the preceding hinge plate residues _261_LV_262,_ the so-called nexus [62,63]. Substitution of the nexus residues, including the Orai1 _261_ANSGA_265_ construct, is able to activate Orai1 fully irrespective of STIM1, thereby indicating the importance of the segment for maintaining the channel in a quiescent state and for gating [62]. Supporting this notion are structural insights on closed and open dOrai channels that point to state-dependent conformational transitions within this region. On the one hand, the M4ext helices from directly neighboring subunits are apparently allowed to interact via coiled-coil packing in the closed state, giving the overall assembly the appearance of a trimer of dimers [43]. In contrast to such inter-subunit associations, the M4ext segments appear to engage a straightened conformation in the crystallographic-resolved open states. Thereby, it needs to be emphasized that all the available presumably open dOrai structures harnessed mutations leading to constitutive, hence STIM1-independent, activity of the channel. Despite decoupled from the physiological activation stimulus, the structurally observed release of interactions between the C-termini of two adjacent Orai subunits might nevertheless serve as a prerequisite for Orai proteins to interact with STIM1 [44]. Consistent with this notion were earlier functional findings of Navarro-Borelly et al. (2008): a decrease in FRET efficiency among C-terminally labelled Orai1 CRAC channel subunits was reported upon co-expression of the Ca^2+^ sensor STIM1 and depletion of stores [35,44,45]. However, extensive straightening as seen in open-state crystals was not observed for the same constitutively active mutant if resolved via cryo-EM. Therefore, it remains to be elucidated if it was indeed superfluous for activation, in particular under physiological circumstances, and if rather subtle changes occurred instead [44]. Further aspects that need to be resolved in this regard are the actual stoichiometry of Orai1-STIM1 binding and an eventual activation stage or condition dependence of it [50,60,63,64,65,66,67].

No matter the question, depletion of the ER as a cell-intrinsic Ca^2+^ store culminates in the opening of the Orai1-formed pore within the plasma membrane. The former specific stimulus is relayed via protein–protein interactions and the interplay/dynamics of the transmembrane helices to the first transmembrane domain and, since composed of TM1 residues, to the hydrophobic and basic gate [62,68,69]. Instead of e.g., α-to-π-helical transitions of the pore-lining transmembrane domain reported for other types of highly Ca^2+^ selective channels to underly pore opening, the α-helicity of the Orai1 TM1 is apparently preserved upon activation. Instead, Ca^2+^ permeation seems to presume some rotation of the pore-lining helix and potentially an increase in hydration of the hydrophobic and basic segments. Furthermore, the latter region eventually undergoes some widening, and the residue R91 gets putatively displaced [51,69,70,71,72].

In addition to the aforementioned consensus on the Orai1 C-terminus as the main site for STIM1 coupling, further domains of Orai1 are crucial within the activation cascade and for a proper CRAC channel function. Considering the remaining cytosolic domains, hence the N-terminus and the loop2 segment, these are predestined to serve as binding platforms for entirely cytoplasmic proteins or for those comprising cytosolic domains, which includes STIM1. Moreover, specific sites therein are apparently involved in the gating process or, at a later stage, the inactivation of the CRAC channel. Importantly, a rather modest overall sequence conservation of 34% of the N-terminal domain and peculiarities within the intracellular loop of the three Orai homologs are also predestined to lead to functional and structural differences. Herein, however, the rather diverse and in some instances controversial role of Orai1 cytosolic domains will be highlighted, while isoform-specific properties of the three Orai proteins were recently recapitulated elsewhere [42].

## 3. Internalization and Recycling

The function of cytosolic domains as interaction sites for various proteins equips these Orai1 regions with a multifaceted and diverse regulatory potential on SOCE and is important for inducing downstream events. On the one hand, interactions between cytosolic portions of Orai1 and proteins of the endocytic machinery orchestrate a continuous internalization and recycling of Orai1 from the plasma membrane under resting conditions. In turn, a rather modest proportion of about 40% of the total protein is available on the surface upon cellular rest [73,74,75]. Moreover, Orai1 internalization is especially promoted during meiosis even though it shows deviations on the molecular level. While normally dependent on Rho- and Rab5 proteins rather than dynamin, endocytosis and recycling are mastered by dynamin, caveolin (Cav) and Rab5 during meiosis. Thereby, a consensus binding site of Cav was found to reside within the N-terminus of Orai1 (aa52–60), involving the aromatic residues Y52 and W55, as indicated in Figure 2 [73,76,77]. Additionally, the Orai1 segment following the fourth transmembrane domain seems to be involved in internalization, whereby the Orai1 span comprising the residues 260-275 was recently termed as the C-terminus Internalization Handle (CIH). Yet, this region is apparently dispensable, as co-expression with Cav was earlier found to re-establish endocytosis of the respective Orai1 deletion mutants (Orai1Δ267-301) [77,78].

Apart from the role of Cav in meiosis-associated internalization, the chaperonin-containing T-complex protein 1 (CCT) chaperonin complex was also found to be relevant for the withdrawal of Orai1 from the cell surface. Protein interactions map in this regard to the loop2 region of Orai1, specifically to the segment interlacing the residues 157 and 167. Consistently, scrambling of the concerning segment was found to lead to increased plasma membrane levels of Orai1. This, in turn, allows for a faster co-clustering with STIM1 upon activation as well as triggering prolonged Ca^2+^ signaling and faster activation of Ca^2+^-dependent transcription factors belonging to the nuclear factor of activated T-cells (NFAT) family [73], which will be discussed in more detail later.

The activation-associated binding between STIM1 and Orai1 interferes with the dynamic internalization procedure. This, in conjunction with the exocytotic machinery, elevates the proportion of Orai1 present in the plasma membrane to up to 65% during cellular stimulation [73,74,75]. Among the proteins promoting, rather than reducing, Orai1 plasma membrane expression is also the synaptosomal-associated protein-25 (SNAP-25), which is a plasma membrane associated SNARE protein [75]. Although the specific interaction site was not determined in the concerning study, it is plausible to assume that it also resides within the cytosolic portions of Orai1. In addition, some isoforms of the secretory pathway Ca^2+^ ATPase of the Golgi apparatus have been supposed as promotors of Orai1 plasma membrane expression, but in a considerably cell type specific manner and primarily in settings where Orai1 proteins mediate store-independent Ca^2+^ entry (SICE) rather than relying on STIM1 dependent activation [79,80,81,82,83].

## 4. Regulation by Lipids

Different groups have suggested that the N-terminus of Orai1 is important for the regulation of CRAC channels by lipids, either directly or indirectly. On the one hand, the latter relates to the presence of protein binding sites within the respective Orai1 segment as well. In this regard, interactions between Orai1 and Cav1, the latter of which was already mentioned to drive plasma membrane withdrawal of Orai1 in the course of meiosis, are apparently again relevant. Yet, Cav1, found to insert itself into the plasma membrane and organelle membranes as a hairpin, was reported to serve as a positive regulator of SOCE in the context of lipidic regulation. This positive effect is eventually explained by the fact that Cav1 delineates a critical scaffolding element of caveolae, i.e., invagination-forming subdomains within the plasma membrane. Apart from structural peculiarities, these environments are characterized by local elevations in cholesterol and sphingolipid levels. Interactions of Cav1 with the N-terminal segment of Orai1 and a section of the terminal transmembrane domain, were stated to drive the accumulation of Orai1 within these peculiar lipid microdomains. While this was subsequently suggested to reinforce the regulatory effect of the enriched lipids on the CRAC channel function [84,85,86,87,88], it should be noted that results on the modulatory impact of cholesterol on Orai1 activity are rather contradictory. On the one hand, Bohórquez-Hernández and colleagues (2017) reported that reductions in cholesterol levels correlate with an increase in the rate of Orai1 internalization, which would indeed be consistent with the aforementioned hypothesis that trapping Orai1 within cholesterol-rich areas fosters activity [84]. On the other, in a study by Derler et al. (2016), cholesterol depletion was found to lead to enhanced SOCE, while elevations of this specific lipid had an inhibitory effect. In the latter study, the N-terminal Orai1 segment _74_LSWRKLYLSR_83_ was identified as matching a prototypical consensus motif for cholesterol interactions [L/V]-(X)_1–5_-Y-(X)_1–5_-[K/R] (Figure 2), where X represents an arbitrary amino acid. The function of this segment as a cholesterol binding locus was supported in this regard by electrophysiological recordings in combination with site-directed mutagenesis and cholesterol depletion efforts. While the latter procedures lacked an effect on CRAC currents of the mutated proteins, cholesterol reduction increased currents of the wild-type control. In addition to this functional analysis, the binding of cholesterol to this region is supported by intrinsic fluorescence-binding studies with Orai1 peptides [89].

Membrane-proximal, cytosolic regions of ion channels are also predestined to be involved in interactions with lipids other than sterols. Positively charged residues within intracellular loops or the C-terminus of various transient receptor potential (TRP) channels were, for instance, identified as relevant for the interaction with phospholipids, in particular with phosphatidylinositol-4,5-bisphosphate (PIP_2_) [90,91,92,93,94]. A regulatory potential of phosphoinositides on SOCE was found as well, irrespective of the need for PIP_2_ cleavage and thereby, IP_3_ generation for activation. Intriguingly, some of the concerning reports even predated the initial description of STIM1 and Orai1 as the molecular players mediating Ca^2+^ entry [95,96]. Considering Orai1, phosphatidylinositol-4-phosphate (PI_4_P) was implicated as a modulator of channel activity. This notion is supported by findings that CRAC currents are sensitive to the inhibition or downregulation of phosphoinositol-4-kinases (PI_4_K), which affects PI_4_P levels. Instead of considerably reducing SOCE upon interference with PI_4_K activity, the depletion of PIP_2_ had no effect in the study by Korzeniowski et al. (2009) on the dependence of CRAC currents on phosphoinositides [97,98]. However, as reviewed in [97], further data are necessary, in particular to shed light on the question of whether this apparent regulatory potential of PI_4_P was indeed based on direct interactions with the channel or if regulation occurred in an indirect manner.

## 5. Activation Gating

Direct interactions between STIM1 and Orai1 are indispensable for transmitting the signal of store depletion to the plasma membrane and finally, to lead to Ca^2+^ entry. In terms of reachability, the cytosolic domains of both proteins––the C-terminus of STIM1 as well as the N-terminus, loop2 domain and the C-terminal region of Orai1––might serve as possible binding sites. While the necessity of the C-terminal coiled-coil domains of both proteins for coupling has extensive experimental and structural support, the role of the N-terminus and the loop2 segment of Orai1 is in this regard still debated [14,44,45,58,59,60,62]. In turn, there are presently two concurrent hypotheses: on the one hand, coupling of the C-terminus of STIM1 to the C-terminal domain of Orai1 is assumed to be sufficient to induce channel opening. On the other, the alternative hypothesis states that additional interactions with the N-terminus and the loop2 segment of Orai1 are needed for gating to occur, eventually occurring in a sequential manner in the activation cascade [29,57,58,62,99,100,101]. Models of the latter kind are for instance backed up by biochemical analysis suggesting that the CAD domain of STIM1 directly associates with the Orai1 N-terminal region aa70-91 [29]. Similarly, McNally et al. (2013) reported that deleting the N-terminal most 85 Orai1 residues or the 73–85 segment diminishes CAD binding by more than 50% [101]. Moreover, there is co-immunoprecipitation data available indicating that the first 48 Orai1 residues would indeed compromise the affinity of CAD for the N-terminus since CAD binding to full-length N-terminal Orai1 fragments was found to be considerably lower than to Orai1_48-91_ [29]. Hydrophobic residues within the final section of the Orai1 N-terminus were specifically reported to serve as interaction sites for the STIM1 residue F394 since after substituting hydrophilic amino acids, activation of Orai1-formed channels was prevented [102]. Yet, considering the lately published data of Niu et al. (2020) on the binding of purified STIM1 fragments to synthetic peptides derived from the Orai1 N- or C-terminus, the interaction with the N-terminus was, in strong contrast to the study of Wang and coworkers (2014), proposed to be hydrophilic and involve the Orai1 residues R77, K78 and L81 [102]. Instead, because coiled-coil domains are involved, coupling with the C-terminus is mainly hydrophobic [103]. Anyhow, awareness is needed that the affinity of STIM1 to the Orai1 C-terminus is generally higher than to the N-terminus. Notwithstanding the latter, interactions among the C-termini of both proteins were recently shown to be lower in the presence of N-terminal Orai1 peptides. This intriguing finding was suggested to indicate that interactions would proceed in a stepwise manner, as anticipated in an earlier paragraph [29,103,104,105].

McNally et al. (2013) found that site-directed mutagenesis within the N-terminus of Orai1 (K85A/E) is capable of reducing both store-depletion triggered interactions with STIM1 and the accumulation of Orai1 within puncta [101]. Instead, others reported that upon co-expression with full-length STIM1, ΔN-Orai1 mutants retained wild-type co-clustering abilities. However, the concerning CRAC channels were inactive, what might serve as indication that the C-terminus does serve as the main binding site for STIM1 but by itself is not sufficient to cause channel activation [14]. This notion is further strengthened by a closer investigation of CRAC channels with disrupted C-terminal STIM1-binding sites. While normally inactive upon co-expression with STIM1, the Orai1 mutants were shown to regain gating abilities if directly fused to specific STIM1 domains. As a result, a stepwise interaction cascade again seems reasonable, with the initial binding of STIM1 to the C-terminus of Orai1 promoting successive interactions with N-terminal loci [106].

Intriguingly, species-specific differences seem to exist in the functioning of the respective Orai cytosolic regions as main interaction platform for STIM1. Recent work of Kim et al. suggests that in the case of *Caenorhabditis elegans*, the loop2 segment serves as the predominant cSTIM1 binding epitope. Furthermore, hydrophobic residues within the intracellular loop that are conserved among the Orai1 proteins of invertebrates but are exchanged by positively charged amino acids in vertebrates, are apparently indispensable for oligomerization and CRAC channel gating. These processes are further reported to rely on intramolecular interactions of the N- and C-terminal domains of *C. elegans* Orai (cOrai) proteins [107]. Indeed, in the case of murine Orai1 (mOrai1) early reports suggest that domains other than the C-terminus serve as *bona fide* binding sites for STIM1 as well; that is, the mOrai1 N-terminus rather than the intracellular loop as was stated for cOrai proteins [37].

Although data on binding to the N-terminus and loop2 are in some respects controversial, it has repeatedly been shown that both segments are critical within STIM1-dependent activation. Considering for instance electrophysiological recordings, CAD was reported to be unable to evoke CRAC currents in cells expressing Orai1 deletion mutants that lack either the complete N- or C-terminal domain or are missing residues 73–84, whereas inducing constitutive Ca^2+^ influx through full-length Orai1 or if Orai1 proteins lack the residues 1–73 [29]. Similar Orai1 regions were also in another study found to be critical for preserving channel activity. The segment Orai1 74–90 is highly conserved among isoforms as well as different species, and it largely coincides with the extended transmembrane Orai1 N-terminal region (ETON, amino acid 73–90, see Figure 2) [14,57].

As might be expected in view of conservation, STIM1-dependent activation of Orai3 was also shown to be sensitive to N-terminal deletions [108]. The N-terminal domain seems nevertheless to contribute to differences in the conduction characteristics of Orai1 and Orai3 because the exchange of the N-terminal segment of Orai3 with that of Orai1 was identified as leading to increased current amplitudes, in addition to accelerating the kinetics. On the other hand, supplementation of Orai1 with the N-terminus of Orai3 considerably compromised store-operated currents [109], a possible explanation for which will be discussed in a later section.

In addition to activation itself, the Orai1 N-terminus and in particular the ETON region (Figure 2) were reported to be critical for the preservation of the typical CRAC channel hallmarks [110]. In this regard, Derler and colleagues (2018) reported that constitutively active Orai1 mutants, including Orai1 L185A and Orai1 P245L, show the characteristic increase in current density upon switching from an extracellular solution containing Ca^2+^ to one that is divalent free, inward rectifying current/voltage (I/V) relationships, inactivation (see later) and comparable reversal potentials as the wild-type CRAC channel only if two basic requirements were met. The prerequisites are co-expression of STIM1 and the presence of an intact Orai1 N-terminus [110]. Consistent with this study, truncations of the N-terminal region were before reported to impede the re-establishment of Ca^2+^ selectivity of the constitutively active Orai1 V102A mutant, which was observed for full-length Orai1 V102A channels upon co-expression with STIM1. Intriguingly, deleting the same region in wild-type Orai1 renders the channel non-functional [57].

While the N-terminally truncated Orai1 V102A preserves activity, withdrawal of the first 78 residues from Orai1 L185A/F250A or Orai1 P245L gives rise to quiescent channels. In contrast to Orai1, the corresponding Δ1-53 Orai3 F160A or Orai3 P254L variants were found to remain active. For the aforementioned analysis of CRAC channel hallmarks, a further modification–the exchange of the Orai1 loop2 segment for that of Orai3–was therefore indeed necessary, resulting in Orai1–Orai3 loop2 chimeras that also maintained activity in the absence of the N-terminus. Similarly, loop2 swapping allowed for the elucidation of current-voltage characteristics and conduction profiles of Orai1 K85E, a mutation which otherwise would interfere with STIM1-mediated activation and further reduces the constitutive activity of a series of Orai1 transmembrane domain mutants [68,109,110]. The Orai1 K85E-Orai3 loop2 chimera was reported to retain comparable channel features as the wild-type, such as the reversal potential and the conduction behavior in a Ca^2+^ free environment. In contrast, the loop2 swap was solely able to restore the activity of Orai1 L74E W76E but was incapable of re-establishing the typical CRAC channel hallmarks. A comprehensive conclusion would thus be that separate regions of the Orai1 N-terminus are required to preserve (I) store-operated activation and (II) CRAC channel hallmarks. While the latter and the fine-tuning of Ca^2+^ influx are dependent on the first half of the ETON region (aa73–79), in particular on residues L74–W76, experimental evidence suggests that the former property relies on the final 10 residues (aa80–90), including K85 [110].

The importance of residue K85 within the cytosolic extension of the pore-lining helix and the role of charged residues in regions of other transmembrane domains that protrude into the cytosol was recently examined closely in a study by Tiffner et al. (2021) [68]. Their analysis of a series of Orai1 double point mutants, each combining one gain-of-function with a loss-of function mutation, suggests that pore opening is orchestrated by local conformational transitions of the transmembrane domains [68]. Indeed, previous studies had also pointed to a concerted interplay of the transmembrane helices as indispensable for unlocking the pore [69,111,112]. In the study of Tiffner et al., extensive functional characterization of the Orai1 double-mutant library indicates that such transmembrane motions spread omnidirectionally through the Orai1 assembly and require a set of gating checkpoints to be passed. The latter seem to be located in the middle of the transmembrane domains yet are also present at their cytosolic extensions. Interestingly, loss-of function mutations mapping onto the cytosolic, extended transmembrane regions were identified to not only impede STIM1 coupling but also to silence constitutively active Orai1 H134A variants, among other gain-of function mutants. Molecular dynamics (MD) simulations and functional analysis indicated that intra (R83-E149, K85-E173)- and inter (K85-E149)-subunit salt bridge formations play a critical activation role by serving as gating checkpoints (see Figure 2). On the one hand, these bridges were found to be partly pre-established at rest yet apparently became fortified in the course of STIM1-dependent Orai1 activation. Although not a premise for STIM1 coupling itself, the salt bridges were nevertheless reported to affect STIM1 binding. This is explained by the observation that the single point mutations K85E or E149K lowered plasma membrane accumulation of C-terminal STIM1 fragments. Instead, plasma membrane targeting was retained upon mutating a pair of salt bridge-forming residues concomitantly in a manner that preserves their interaction abilities (Orai1 K85E E173K or Orai1 R83E E149K). Altogether, the gating checkpoints seem to be pivotal for the Orai1 assembly to engage an integer pore geometry and to establish an opening-permissive conformation [68]. Interestingly, 10 residues subsequent to the mentioned salt-bridge forming E149, the Orai1 S159L mutant has been identified to own clinical relevance. The mutation is associated with uterine carcinoma and, according to Frischauf et al. (2017), renders the channels constitutively active upon expression in human embryonic kidney cells [69,113].

Despite full conservation of the ETON region in Orai1 and Orai3 (aa48–65), the latter tolerates the deletion of larger segments than Orai1 to nevertheless retain STIM1-dependent activation. However, this seems to be primarily dependent on the peculiarities of the respective loop2 segments rather than the N-terminus itself. In this regard, the remaining N-terminal portion of the Orai1 loss-of function deletion mutants apparently engage interactions with the loop2 region, thus leading to inhibition. In strong contrast, such associations are absent in the equivalent Orai3 N-terminally truncated variants. Although the loop2 stretches of Orai1 and Orai3 show a rather high sequence identity of up to 75%, MD simulations revealed the TM2 helix of Orai3 to be longer, resulting in a shorter, less-flexible intracellular loop. Introduction of five mutations within the Orai1 loop2 to mimic the respective cytosolic domain of the Orai3 homolog was shown to suffice for restoring store-dependent activation of Orai1 Δ1-78. Conversely, however, the exchange of the Orai3 loop2 by that of Orai1 silenced otherwise active N-terminal deletion Orai3 constructs [99]. Also in the case of full-length Orai1, an association of the loop2 segment with the N-terminus seems to have a negative effect on CRAC channel activity. Indeed, the forced close proximity between the N-terminal domain and the intracellular loop, achieved by the cysteine crosslinking of K78C and E166C, was unraveled to interfere considerably with store-dependent activation of full-length Orai1. Moreover, co-localization with cytosolic STIM1 fragments was reported to be increased for Orai1 Δ1-78 upon swapping of the loop2, yet still remained inferior compared to the levels reached with wildtype Orai1. In this regard, it is tempting to speculate that the inhibitory effect of the Orai1 loop2 segment was due to some interference with STIM1 coupling and is further supported by the N-terminal segment of the ETON region, whereby it needs to be elucidated if this occurs directly or allosterically [99,110]. Taken together, this data explains, at the molecular level, the need for loop2 swapping in the afore mentioned study on CRAC channel hallmarks as well [99,110].

Recent data indicate that the interplay between a short STIM1 segment (STIM1 α3, amino acids 400–403) and the cytosolic extension of the third transmembrane domain of Orai1, creating a STIM1–Orai1 gating interface (SOGI; indicated in Figure 2), was critical for activation gating. Although binding to Orai1 was reported to be preserved upon site-directed mutagenesis of STIM1 residues within this region or upon deleting the whole segment, CRAC current activation was abolished. Hence, close proximity of the cytosol reaching TM3 segment of Orai1 and STIM1 α3, shown in cysteine crosslinking experiments, seems to be a premise for STIM1 coupling to be transduced into gating the Orai1 pore rather than representing a main STIM1 binding site [114].

## 6. Stabilization of the STIM1/Orai1 Complex

There is growing evidence that the formation and maintenance of the CRAC channel complex upon cellular activation relies on a series of accessory proteins rather than being exclusively mastered by STIM1 and Orai1. Indeed, possible interaction sites have been identified throughout the entire sequences of both STIM1 and Orai1. In this regard, the cytosolic N-terminus and particularly the C-terminus of Orai1 were found to be relevant for interactions with the soluble protein α-SNAP. Upon store depletion, interactions of α-SNAP with both basic components of the CRAC channel complex seem to be a critical determinant for the molecular composition of STIM1-Orai1 clusters. Indeed, ablation of α-SNAP was reported to reduce SOCE and the nuclear translocation of NFAT in a manner correlating with an increase in the per-cluster density of Orai1. Given that the density of STIM1 was at the same time unaffected, this implies a change in the proportion of Orai1 channels that co-cluster with STIM1. Thus, this specific accessory protein was suggested to be critical for the maintenance of appropriate STIM1/Orai1 ratios upon interaction, thereby activating SOCE in a physiological range [115]. Later, however, α-SNAP was reported to orchestrate the on-site assembly of resting-state dimeric Orai1 into multimers of the characteristic Ca^2+^ selectivity after the depletion of ER stores. Moreover, depletion of this interaction partner apparently allowed for a higher mobility of Orai1 proteins co-clustering with STIM1 at ER–PM junctions in the concerning study. Despite lacking an effect on FRET between STIM1 and the C-terminus of Orai1, a compromised energy transfer between STIM1 and the N-terminus of Orai1 was monitored by Li and coworkers (2016) as well. In turn, α-SNAP was proposed to drive conformational adaptions of the N-terminus that were critical for a firm association with STIM1 and would consequently allow for the assembly of Orai1 into higher-order oligomers to give rise to a Ca^2+^ selective pore [116]. However, this study on the on-site assembly of Orai1 dimers into the actual oligomeric channel upon store depletion seems to be difficult to reconcile with structural data on dOrai-formed channels and with other functional studies [46,47].

During the preparation of this review, another study targeting the role of α-SNAP in CRAC channel activation was released. Among other Orai1 residues of the N-terminus and the hinge region, L74 was reported to be critical for interactions with α-SNAP after store depletion. In the concerning study, Ramanagoudr-Bhojappa et al. (2021) suggested that high affinity interactions between the latter protein and Orai1 but also STIM1 were critical for functional SOCE to arise, yet the interplay was nevertheless stated to be dispensable for STIM1–Orai1 co-clustering [117].

Further lines of evidence indicate that the N-terminus of Orai1 is critical for the formation of stable STIM1–Orai1 complexes upon store depletion by presenting a binding platform for cytosolic proteins. Among these rank the homologous Ca^2+^-sensing proteins CRACR2A (CRAC regulator 2A) and CRACR2B. In view of a study by Srikanth et al. (2010), the modulators function as interaction partners of both STIM1 and Orai1 as well. These proteins, which own Ca^2+^ sensing abilities, were reported to be critical for clustering and by forming a ternary complex with STIM1 and Orai1, they presumptively stabilize the interaction of the CRAC channel proteins [118]. In the case of Orai1, the basic residues K85 and K87 seem to be vital for interaction with this regulator [118]. Yet, it needs to be stressed out that these sites reside rather deep within the pore, what rises concerns about their availability for interactions to occur [119]. Apart from reachability itself, protein trapping at this location might be problematic for ion conduction.

Neglecting these concerns, the association of CRACR2A with the CRAC channel complex was reported to occur in a Ca^2+^ sensitive manner. Specifically, only under conditions of low cytosolic Ca^2+^ levels that deprive the EF-hand domains of CRACR2A of the ion, the particular modulator was found to interact readily with STIM1 and Orai1. This implies that the accessory protein is associated early in the activation process, while interactions with the CRAC channel complex disappear in further sequence when Ca^2+^ levels rise. Further supporting Ca^2+^ dependence, mutations within the EF-hand domains of CRACR2A were reported to lead to elevations in the basal Ca^2+^ levels upon expression in T-cells. Moreover, this was associated with a concomitant increase in the rate of cell death.

In addition, the interaction of Cav1 with STIM1-complexed Orai1 was said to foster stability of the CRAC channel complex thereby enhancing SOCE [88].

## 7. Induction of Downstream Responses

Apart from protein trafficking, stabilizing STIM1/Orai1 interactions and activation gating, cytosolic Orai1 domains contribute to the induction of downstream events as well, such as the activation of Ca^2+^-dependent transcription factors. In this regard, awareness is needed that the versatility in the responses of processes harnessing Ca^2+^ as an intracellular messenger is ensured by Ca^2+^ signals showing a considerable variety in their duration (transient to stable) and oscillation patterns. Moreover, there is some cell-type specificity in the expression patterns of the involved proteins. Apart from differences in the temporal dimension, whether the concentration of free intracellular Ca^2+^ increases globally or in a spatially restricted fashion tailors Ca^2+^ entry to the stimulation of distinct downstream events [1,2,4,5,6]. In the case of Orai1, the extraordinary low single-channel conductance within the range of 10–30 femto-Siemens (fS) needs to be emphasized in this regard. Indeed, this characteristic predestines CRAC channels to give rise to increases in the Ca^2+^ concentration that are locally restricted to the vicinity of the open pore rather than supporting a pronounced heightening in the overall cytosolic Ca^2+^ level [120,121]. Importantly, this quality tailors CRAC channels to activate specific transcription factors, including NFAT1 [121,122,123]. The group of Ca^2+^-regulated NFAT proteins comprises four members (NFAT1-4), which are retained within the cytosol under resting conditions due to extensive phosphorylation of several sets of serine- and threonine-rich segments. Specifically, the trapping in the cytosol at rest is based on the association of the negatively charged phosphate moieties with basic residues serving as nuclear localization signal (NLS). After cellular stimulation, dephosphorylation of NFAT and its consequent conformational adaptions allow for nuclear import and transcriptional activation. Thereby, Ca^2+^ dependence of this simplified activation process relates to the ubiquitous Ca^2+^ sensor protein calmodulin (CaM), which stimulates the phosphatase calcineurin in the presence of Ca^2+^ [124,125,126,127,128].

The ability of such multi-staged activation cascades to respond to Ca^2+^ micro- or even nanodomains, whereby increases in the Ca^2+^ concentration reach several nanometers below the inner leaflet of the plasma membrane, relies again on the presence of protein binding epitopes within the Orai1 cytosolic regions. In this regard, a section of the Orai1 N-terminal domain is relevant, as it owns affinity for the scaffolding protein A-kinase anchoring protein 79 (AKAP79) (Figure 2). The latter concomitantly tethers the phosphatase calcineurin and inactive NFAT transcription factors [129]. While the Orai1–AKAP79 interaction is weak in the absence of stimulation, it is promoted upon activation. Together with CaM, which is also eventually bound to the N-terminus of Orai1, this gives rise to a signaling complex that fosters dephosphorylation of NFAT by calcineurin. The most N-terminal 89 Orai1 residues seem to be critical in this regard since their deletion was found to hinder AKAP79 binding and interfere with NFAT1 activation. Specifically, an analysis of a series of Orai1 deletion mutants suggests that the AKAP association region (AKAR) involves the Orai1 residues 39 and 59 [42,129,130,131]. Interestingly, the use of an alternative translation initiation site -methionine 64 of the longer Orai1α variant–yields a shorter Orai1 isoform, Orai1β, which consequently deviates in the N-terminal domain and AKAP binding. This in turn curtails the ability of the shorter Orai1 version to drive NFAT activation. Similarly, lacking conservation of the concerning segment within the N-termini of both, Orai2 and Orai3 proteins accounts for differences in their transcription factor activation abilities [42,130,131].

Apart from AKAP79, the concomitant interactions of Cav1 as scaffolding protein with Orai1 and downstream-acting signal transducers are thought to give rise to signaling hubs. Thereby, the activation of different Ca^2+^ dependent transcription factors was reported to be specifically and reciprocally regulated in dependence of the precise Ca^2+^ signal and separate Cav1 domains, respectively. In turn, local Ca^2+^ entry via the Orai1 pore, i.e., the common trigger for NFAT but also c-Fos-dependent gene expression, may be tunneled down to a specific pathway to activate one type of transcription factor but not the other [88].

## 8. Ca^2+^ Dependent CRAC Channel Inactivation

Stringent control over the timing and extent of Ca^2+^ influx is vital for the induction of divergent cellular responses, for a proper cell function and not least, for cell survival [132,133]. Apart from regulatory mechanisms launching at the level of activation gating, the modulation of ion currents and channel deactivation if the ER-luminal Ca^2+^ reservoir has been replenished, SOCE is adjusted via processes of Ca^2+^ dependent inactivation [134]. Considering the latter, the entering Ca^2+^ ions itself serve as a signal to drive the abandonment of further influx via negative feedback processes. Neglecting the actual mechanisms, Ca^2+^-dependent inactivation (CDI) is not only a feature of Orai1 and its homologs but is a prevalent characteristic of also other types of Ca^2+^-selective channels, such as voltage-gated Ca^2+^ channels or the Ca^2+^-selective members of the transient receptor potential vanilloid-type (TRPV) subfamily, TRPV5 and TRPV6 [18,132,135,136,137,138,139,140,141].

Ca^2+^-dependent inactivation of CRAC channels occurs on two different time scales: fast Ca^2+^-dependent inactivation (FCDI) happens on the order of milliseconds, whereas slow Ca^2+^-dependent inactivation (SCDI) needs 10 of seconds to develop [133]. While a comprehensive overview of both inactivation processes with all their unresolved questions is beyond the scope of this review, there is again experimental support that the Orai1 N-terminus as well as the intracellular loop would be indispensable in these regards. On the one hand, calmodulin (CaM) was found to preferentially associate with N-terminal segments of Orai1 only in the presence of Ca^2+^, which raised suggestions that this ubiquitous Ca^2+^ binding protein accounted for the Ca^2+^ dependence of the inactivation process [142,143]. Indeed, expression of calmodulin-inhibitory peptides or specific calmodulin variants hampered in Ca^2+^ binding abilities was observed as correlating with reductions in the level of fast inactivation [142,144]. Moreover, mutagenesis within the calmodulin-binding domain of Orai1 (CBD_Orai1_) to impede interactions with calmodulin was shown to compromise FCDI. Yet, structural insights into dOrai-formed channels again raised concerns about whether the peculiar Orai domain involving the Orai1 residues 68–91 might truly be engaged by CaM. Especially the side chains of the residues W76 and Y80, reported to be critical for CaM binding, were found to face the rather narrow CRAC channel pore, which is speculated to preclude the association of the regulatory protein for steric reasons [142,143] and was analogously discussed for another regulator in a former section. However, X-ray crystallographic data on isolated CaM-binding peptides of Orai1 in complex with CaM eventually alleviate such doubts raised by dOrai structures. This is explained by the Ca^2+^ sensor accommodating a rather uncommon, extended conformation in this association. Interestingly, only the C-terminal lobe of CaM appeared to interact with the respective Orai1 segment. In concert with isothermal titration calorimetry, pulldown experiments and gel filtration chromatography, this led to the hypothesis that CaM drives inactivation of Orai1 in a two-step process, commencing with the association of the C-lobe of CaM to the CBD_Orai1_. This in turn led to the binding of the N-terminal lobe to the CBD_Orai1_ of a neighboring Orai1 subunit, according to the theory of Liu et al. (2012) [143]. Such a stepwise binding mechanism was later further supported by extensive data of Traxler et al. (2017), who were further able to quantify an exceptionally high affinity of CaM binding to a pair of CBD_Orai1_ [145]. Additionally, the same efforts identified conservation of such highly affine interactions in the case of dOrai proteins [145]. Moreover, a recent thermodynamic evaluation of Maganti et al. (2019) suggests that the association of the C-terminal CaM domain with Orai1 allosterically affects the N-terminal lobe [146].

As an alternative to CaM binding, Mullins et al. (2016) proposed that the mere presence of sterically demanding, hydrophobic W76 and Y80 side-chains within the pathway of ion conduction was important for functional inactivation [147]. Also, the deployment of M64 as an alternative translation initiation site affects FCDI, as channels composed of the truncated Orai1β variant support FCDI to a reduced extent only [148]. Considering a recent study by Zhang et al., this might again relate to differences in the binding abilities of AKAP79, in analogy to what was stated in the context of NFAT activation. In this regard, however, the Ca^2+^-activated adenylyl cyclase 8 (AC8) was found to bind constitutively to a triad of arginine residues (R31, R32, R33) in the N-terminal segment of Orai1. Thereby, the enzyme gets positioned in the immediate vicinity of the Ca^2+^ permeating pore [149,150]. Upon gating into the open state, entrant Ca^2+^ ions drive the activation of AC8 and followingly, the generation of cyclic adenosine monophosphate (cAMP). The latter metabolite in turn activates protein kinase A (PKA), which is tethered to Orai1 via AKAP79, so that Orai1 gets phosphorylated at S34, which is stated to promote inactivation [150,151]. Intriguingly, concurrent activation of also calcineurin antagonized phosphorylation by PKA. This results in a sophisticated interplay of phosphorylation and dephosphorylation to shape the cytosolic Ca^2+^ signals in magnitude and duration. As mentioned in an earlier section, the stimulation of different downstream events is thereby forced. Indeed, NFAT isoforms also respond to different spatiotemporal Ca^2+^ signals and become separately activated, yet the extent of their activation is modulated by the precise characteristics of the Ca^2+^ stimulus either [150,152].

Apart from PKA-dependent phosphorylation of S34, others reported that protein kinase C (PKC)-mediated phosphorylation of the serine residues S27 and S30 negatively modulates SOCE by promoting inactivation [153]. Intriguingly, phosphorylation of these serine residues was found to be hindered upon overexpression of AC8, given the immediate proximity and an eventual partial overlap of the binding site of the cyclase and the loci to be modified. This might comprise clinical relevance, considering that the breast cancer cell lines MDA-MB-231 and MCF7 reveal elevated expression levels of AC8 and Orai1 itself in comparison with a cell line derived from non-tumoral breast epithelium [154].

In addition to the former findings, an intact communication of STIM1 with the N-terminus of Orai1 was reported to be a premise for an integer inactivation although it remains to be determined if this involved direct binding or was allosteric in nature. This is explained by data on the constitutively active Orai1 P245L mutant, which lacks inactivation exclusively in the absence of STIM1. However, deletion of the N-terminal domain of this mutant was identified to ablate FCDI in both the presence and absence of STIM1. While this ΔN-Orai1-P245L construct retained constitutive activity in the concerning study, ΔN-Orai1 variants were inactive upon co-expression of STIM1 [110]. Moreover, the N-terminus of Orai1 was reported to mediate reactivation subsequent to FCDI, relying on proline/arginine-rich segments therein [155].

In addition to such multifaceted roles of the N-terminus, the loop 2 segment of Orai1 was also implicated in fast channel inactivation. Analogous to well-established inactivation mechanisms of other types of ion channels, whereby inactivation is commonly mediated by the physical occlusion of the ion conduction pore by cytosolic domains of the channel protein via ball-and-chain or hinged-lid mechanisms, the intracellular Orai1 loop was supposed to act as blocking particle [156,157,158,159]. In support thereof, FCDI was found to be sensitive to mutations of the loop residues Orai1 _151_VSNV_154_. Conversely, CRAC currents were blocked upon supplementation of peptides comprising either the sequence of the entire intracellular loop or just the _153_NVHNL_157_ motif. Further, analysis of concatemeric Orai1 wild-type or loop-mutant constructs indicated that a single wild-type loop 2 within the overall channel assembly was sufficient to restore fast inactivation. Without going into detail, Ca^2+^ dependence of such a physical pore occlusion was again supposed to rely on CaM as a sensor protein [156].

CRAC channels subserve slow inactivation processes as well, but SCDI, which is modulated by the uptake of Ca^2+^ into mitochondria, seems to be primarily orchestrated in a STIM1-dependent manner and thus is not discussed herein [160,161,162,163].

## 9. Regulation by Cellular Factors

In addition to the regulatory potential upon serving as an interaction platform for modulatory proteins, both the N-terminus and the loop2 of Orai1 were also found to be responsible for the dependence of CRAC channel activity on pH and on redox conditions. Specifically, the current amplitude and inactivation kinetics of Orai1 apparently depend on internal pH. In experimental settings, intracellular pH may be modified by adjusting the pipette solution in electrophysiological recordings conducted in the whole cell mode or by the addition of sodium propionate or ammonium chloride to the bath solution for acidification and alkalinization, respectively. Irrespective of the strategy of manipulation, lowering of the intracellular pH reduces I_CRAC_. Instead, a shift towards higher pH values goes along with a current increase. FCDI, on the other hand, is negatively affected by both acidification and alkalinization. In addition to inactivation, reactivation is reduced if intracellular pH is raised, whereas decreases in pH correlate with an increase in reactivation. In contrast to Orai1, Orai3 is not sensitive to changes in intracellular pH. Thereby, experiments on Orai1–Orai3 chimeras provide strong evidence that the N-terminus and the loop2 would be responsible for the pH dependance of Orai1 [164,165]. Intriguingly, in the case of Orai2, only the current amplitude was reported to be sensitive to intracellular pH [164].

The ratio of STIM1 and Orai1 expression levels was early found to affect I_CRAC_ in terms of maximum current, FCDI and Ca^2+^-dependent reactivation at negative potentials. Based on these findings, the hypothesis was raised that the pH-dependence of Orai1-related I_CRAC_ would indeed go back to a regulatory function of intracellular pH on the STIM1-Orai1 interplay [65,164,166]. FCDI is well established to be promoted in the case of comparatively low Orai1–STIM1 expression ratios, while reactivation is increased if STIM1–Orai1 ratios are low [65,164,166]. This, together with data that the N-terminus and the cytosolic loop of Orai1 possess an affinity for STIM1, recently led Rychkov et al. (2020) to speculate that changes in intracellular pH would alter the protonation state of residues within these Orai1 segments and thereby, protein-protein interactions. [164]. In line with this notion, the Gill lab showed recently that STIM1–Orai1 binding is rapidly inhibited under hypoxic conditions, the latter of which provokes cytosolic acidification [167]. While a closer characterization of the Orai1 loci that were relevant in this regard remained elusive in the former study, others found that the loop2 residue H155, which is conserved among the three Orai isoforms, was pivotal for intracellular pH sensing. This is explained as its substitution by phenylalanine apparently ablated responses to both acidification and alkalinization [167,168].

Altogether, there is growing evidence for the pH sensitivity of CRAC channels to be accomplished in a multifaceted manner, considering that the pH-dependence of both the reactivation and the current amplitude was found to be affected by point mutations of STIM1 as well, but in a different manner [165].

Apart from intracellular pH, CRAC channel function is further affected by acidosis or alkalization within the extracellular milieu [168,169]. Although a closer discussion of the underlying mechanisms shall be neglected herein, it should be emphasized that pH sensitivity of CRAC channel activity holds critical physiological functions and is highly relevant under pathophysiological conditions as well [168].

In addition to pH, Orai1-formed channels further experience redox regulation. Thereby, the Orai1 residue C143 that is located at the beginning of the loop2 segment seems to be involved. Yet, despite contributing to regulation, its relevance is apparently low compared to that of the extracellular residue C195 (Figure 1). Intriguingly, CRAC channel activity in different types of lymphocytes, which face considerably oxidizing milieus in the course of inflammation, was identified to be affected differently by H_2_O_2_. Thereby, oxidation was reported to inhibit Orai1 function if expressed in naïve T helper cells, whereas redox-sensitivity was reported to be progressively reduced upon differentiation of effector cells [56].

## 10. Conclusions and Outlook

Calcium release-activated calcium (CRAC) channels represent a pivotal pathway for Ca^2+^ to enter the cytosol from the extracellular space, which in turn drives diverse cellular responses, is responsible for re-establishing Ca^2+^ homeostasis and for mediating different physiological processes. Thus, the identification of its basic molecular components—the proteins STIM1 and Orai1—was a major breakthrough in the field of Ca^2+^ signaling and kicked off extensive structural and functional characterization thereof. Thanks to these efforts, it is now well established that depletion of the ER as internal Ca^2+^ store triggers STIM1 activation, involving conformational rearrangements, aggregation and translocations to prepare for direct interactions with Orai1, leading to pore opening in final consequence [11,12,13,14,15,42,170]. In turn, much attention has been paid to elucidating the interaction among these proteins, showing that coiled-coil domains in the C-termini of the proteins doubtless play an essential role. However, less well-resolved are the functions of the remaining cytosolic Orai1 portions: the N-terminus and the loop2. Despite room for further study, there is, as reviewed in the present work, already solid experimental evidence that the N-terminus and loop2 of Orai1 are important for CRAC channel regulation and are altogether critical for activation. On the one hand, both domains contain binding sites for entirely cytosolic or membrane-associated proteins that orchestrate surface expression of Orai1 and endocytosis, trap the pore-forming protein in specialized subregions of the plasma membrane, regulate the ratio of STIM1 and Orai1 interacting with one another upon cellular stimulation, stabilize the CRAC channel complex or scaffold signaling hubs [73,76,77,85,86,87,89,115,118,129]. Moreover, the N- and C-terminal regions of Orai1, as well as some pore residues have been identified as interaction sites for the Ca^2+^-activated K^+^ channel SK3. Instead of SK3 serving as a regulator for CRAC channel activity, the interplay with Orai1 enhances the activity of the specific potassium channel. Indeed, Orai1 expression was recently found to be able to restore the activity of SK3 upon overexpression of Ca^2+^-insensitive CaM mutants [171]. Anyhow, the N-terminus of Orai1 is, in concert with STIM1, indispensable for CRAC currents to develop their characteristic features as well, and the cytosolic extensions of the membrane-spanning domains comprise gating checkpoints vital for the opening of the pore [57,69,110]. Cytosolic regions of Orai1 have been identified as lipid binding sites, in addition to an apparent relevance of N-terminal regions and sections of the second loop for fast Ca^2+^-dependent inactivation, among others [95,96,97,142,143,149,150]. Notwithstanding the latter, as was emphasized in this review, a consensus on how CRAC channel inactivation is orchestrated is still missing. While refraining from recapitulating an earlier section, it should in this regard be emphasized that doubts on a specific hypothesis, as in the case of CaM binding abilities, might be alleviated to some extent by further analysis under appropriate conditions [119,143,144,145].

Deeper mechanistic insights are not only in the latter case still awaited. Instead, it needs to be underscored that there are still many open questions concerning the multifaceted functions of the Orai1 N-terminus and the loop2 domain on the molecular/mechanistic level. In particular, their role as interaction sites for STIM1 and associated questions like the stoichiometry of binding need to be revisited, given that the STIM1–Orai1 interplay is altogether essential for CRAC channel activation. There is also plenty of room for close-up studies on Orai1 regulation by lipids, for instance, to resolve the controversy over whether cholesterol serves as positive or negative modulator, as well as to determine the role of phospho- and other lipids [84,89,98].

Considering that the malfunction of CRAC channels has been associated with various diseases including different types of cancer, these domains might be further implicated as important drug targets [41,172,173]. This is particularly relevant in the case of gain-of function mutations or pathological increases in expression levels, whereby treatment with CRAC channel blockers was desirable [174]. As listed in [175], a series of agents affecting CRAC currents has already been presented. Yet, their actual clinical use is bedeviled by concerns about a lack of specificity, low efficacy and the widespread expression of STIM1 and Orai1 in various cell types, which is on danger to give rise to side effects. As a consequence, the list of inhibitors that actually have entered clinical trials is sparce. Notwithstanding what was stated before, recent characterization of the blocker Synta66 revealed that the sensitivity of vascular smooth muscle was considerably higher in comparison with immune system cells so that tissue-selective blocks might indeed be achieved [174,176]. To sum up, it is tempting to speculate that a closer characterization of the herein discussed Orai1 domains and the relevance of different sections as protein–protein interaction sites or their involvement in different stages of activation gating and inactivation might not only benefit a deeper understanding of a normal CRAC channel function but might also pioneer further drug development.
